# The impact of adiposity on adipose tissue-resident lymphocyte activation in humans

**DOI:** 10.1038/ijo.2014.195

**Published:** 2014-12-23

**Authors:** R L Travers, A C Motta, J A Betts, A Bouloumié, D Thompson

**Affiliations:** 1 Department for Health, University of Bath, Bath, UK; 2 Unilever R&D Vlaardingen, Vlaardingen, The Netherlands; 3 INSERM U1048, Institut des maladies métaboliques et cardiovasculaires and Université Paul Sabatier, Toulouse, France

## Abstract

**Background/objectives:**

The presence of T lymphocytes in human adipose tissue has only recently been demonstrated and relatively little is known of their potential relevance in the development of obesity-related diseases. We aimed to further characterise these cells and in particular to investigate how they interact with modestly increased levels of adiposity typical of common overweight and obesity.

**Subjects/methods:**

Subcutaneous adipose tissue and fasting blood samples were obtained from healthy males aged 35–55 years with waist circumferences in lean (<94 cm), overweight (94–102 cm) and obese (>102 cm) categories. Adipose tissue-resident CD4+ and CD8+ T lymphocytes together with macrophages were identified by gene expression and flow cytometry. T lymphocytes were further characterised by their expression of activation markers CD25 and CD69. Adipose tissue inflammation was investigated using gene expression analysis and tissue culture.

**Results:**

Participants reflected a range of adiposity from lean to class I obesity. Expression of CD4 (T-helper cells) and CD68 (macrophage), as well as FOXP3 RNA transcripts, was elevated in subcutaneous adipose tissue with increased levels of adiposity (*P*<0.001, *P*<0.001 and *P*=0.018, respectively). Flow cytometry revealed significant correlations between waist circumference and levels of CD25 and CD69 expression per cell on activated adipose tissue-resident CD4+ and CD8+ T lymphocytes (*P*-values ranging from 0.053 to <0.001). No such relationships were found with blood T lymphocytes. This increased T lymphocyte activation was related to increased expression and secretion of various pro- and anti-inflammatory cytokines from subcutaneous whole adipose tissue explants.

**Conclusions:**

This is the first study to demonstrate that even modest levels of overweight/obesity elicit modifications in adipose tissue immune function. Our results underscore the importance of T lymphocytes during adipose tissue expansion, and the presence of potential compensatory mechanisms that may work to counteract adipose tissue inflammation, possibly through an increased number of T-regulatory cells.

## Introduction

The presence of immune cells within human adipose tissue was only discovered a decade ago when macrophages were first identified in abdominal subcutaneous adipose tissue.^1^ Since then, macrophages have taken centre stage in this field of research,^2, 3, 4, 5^ whereas the roles of lymphocytes and other cells of the immune system have been largely overlooked in human studies. This is despite the fact that lymphocytes are key players in the initiation and regulation of immune responses within conditions such as atherosclerosis, asthma and rheumatoid arthritis.^6, 7, 8^ Most investigations of T lymphocytes in human adipose tissue have used immunohistochemistry, gene expression and/or flow cytometry to identify the presence of these cells.^9, 10, 11, 12, 13, 14, 15^ Indeed, other than the documented presence of activated T lymphocytes in adipose tissue,^10, 16^ little is known about the extent of lymphocyte activation and whether this is influenced by levels of adiposity and adipose tissue function.

Studies using rodent models of diet-induced obesity to investigate the time course of immune cell accumulation in adipose tissue suggest that T lymphocytes precede macrophage infiltration/proliferation^12, 15^ and that cytotoxic (CD8+) lymphocytes in particular may be key mediators of early adipose tissue inflammation, insulin resistance and macrophage migration, activation and differentiation.^15^ These animal models provide extremely useful insights but require confirmation in humans, especially since obesity typically occurs over a much longer period than diet-induced obesity in rodents. Importantly, these rodent studies reinforce the hypothesis that lymphocytes have a central physiological role during the early stages of adipose tissue expansion. Much of the available information about immune cells in human adipose tissue comes from morbidly obese individuals where the state of dysfunction is already profoundly apparent at a systemic level, characterised by insulin resistance/type 2 diabetes with adipose tissue showing a pro-inflammatory phenotype.^10, 11, 12, 14^ Less is known, however, about the role of adipose tissue-resident immune cells during more ‘common' forms of overweight and obesity. This is important from a mechanistic and clinical perspective as ~62% adults in the United Kingdom are overweight with <3% of people having a body mass index >40 kg m^−2^.^17^ Additionally, interventions targeting people with modest obesity and ‘early' metabolic dysfunction would yield the greatest rewards given the relative prevalence of modest obesity and the opportunity to prevent the development of future adiposity-related chronic disease.

Thus, the aim of the present study was to investigate human adipose tissue-resident T lymphocyte subsets and their activation in lean to moderately obese individuals who have been carefully characterised in terms of metabolic health. Furthermore, to put these findings into context, we also determined whether T lymphocyte activation was related to either pro- or anti-inflammatory properties of adipose tissue and to commonly used clinical markers of metabolic health.

## Materials and methods

### Experimental design

Thirty men aged between 35 and 55 years were recruited by local advertisement and visited the laboratory for preliminary anthropometric measurements including waist circumference, which was used to classify participants as lean <94 cm, overweight >94 cm but <102 cm and obese >102 cm.^18^ Recruitment continued until there was an equal distribution of 10 participants in each waist circumference category. Participants also attended the laboratory on one separate occasion for sampling of blood and adipose tissue. Blood samples were collected for measurement of clinical markers of metabolic health and isolation of peripheral blood mononuclear cells (PBMCs). Adipose tissue samples were divided into three portions for gene expression analysis, culture or isolation of the stromavascular fraction (SVF). The protocol was reviewed and given approval by the South West, Southmead NHS Research Ethics Committee (REC Reference: 11/SW/0193) and all participants provided written informed consent.

### Participants

Participants were grouped according to waist circumference and individuals were excluded if they had any medical conditions or were taking any medication known to interfere with immune function or lipid/glucose metabolism. Individuals were also excluded if they smoked or had not been weight stable for more than 3 months (i.e., weight change >3%).

### Pretrial requirements

Participants were asked to refrain from performing any strenuous physical activity and consuming alcohol/caffeine for 48 and 24 h before testing, respectively. Trial days were scheduled so that participants had been free from any self-reported illness for a minimum of 2 weeks to reduce immune system disturbance. Participants arrived in the laboratory in the morning after an overnight fast (minimum 10 h) and consuming 1 pint of water upon waking and rested in the laboratory for 45 min before testing.

### Body composition analysis

Body mass (post-void on the morning of the trial) was determined with participants wearing lightweight shorts using a digital balance (Tanita Corp., Tokyo, Japan). Body composition analysis was performed using dual-energy X-ray absorptiometry (Discovery; Hologic, Bedford, UK). Central adipose tissue was estimated from a central region between L1 and L4, which has previously been shown to correlate with measures of metabolic health.^19^ Fat mass index (FMI) was calculated using the equation: FMI=total fat mass (kg)/height^2^ (m^2^) and interpreted using the ranges that match the World Health Organisation Body Mass Index classifications.^20^

### Blood and adipose sampling and preparation

A venous blood sample was taken from an antecubital vein and dispensed into tubes containing either K3EDTA or serum separation beads (Sarstedt Ltd, Leicester, UK). Samples for plasma separation were immediately centrifuged at 3465 *g* for 10 min at 4 °C. Serum samples were left to clot for 45 min before centrifugation. PBMCs were isolated by density gradient separation (Lympholyte; Cedarlane Laboratories Ltd, Burlington, ON, Canada). PBMC pellets were stored in 1 ml phosphate-buffered saline:foetal bovine serum:dimethyl sulphoxide (5:4:1) and frozen at a rate of −1 °C min^−1^ using a freezing container (Nalgene; Thermo Scientific, Waltham, MA, USA) to −80 °C and stored before subsequent analysis by flow cytometry.

Subcutaneous adipose tissue samples (~1 g) were obtained under local anaesthetic (1% lidocaine) ~5 cm lateral to the umbilicus with a 14 G needle using an ‘aspiration' technique.^21^ Visible connective tissue, blood and clots were removed from the adipose tissue with scissors before the remaining adipose tissue was washed with phosphate-buffered saline over single-use sterile gauze membrane to remove any further blood, clots and connective tissue. Approximately 200 mg whole adipose tissue was transferred to an RNase/DNase-free sterile centrifuge tube and frozen immediately on dry ice and later homogenised in Trizol (Invitrogen, Paisley, UK). The remainder was used for adipose tissue culture, or preparation of SVF as described below.

### Adipose tissue culture

Small portions of adipose tissue were minced (~5–10 mg) using sterilised scissors and transferred to sterile culture plates (Nunc, Roskilde, Denmark) containing endothelial cell basal media supplemented with 0.1% fatty acid-free bovine serum albumin and 100 U ml^−1^ penicillin and 0.1 mg ml^−1^ streptomycin (Sigma-Aldrich, Gillingham, UK). Tissue was incubated at a final concentration of 100 mg tissue per 1 ml in duplicate^22^ at 37 °C with 5% CO_2_ and 95±5% relative humidity (MCO-18A1C CO_2_ incubator; Sanyo, Osaka, Japan). After 3 h, media were removed and transferred to sterile eppendorfs and stored at −80 °C.^22^

### Preparation of SVF

The remaining tissue was digested using type I collagenase (Worthington Biochemical, Lakewood Township, NJ, USA) at 250 U ml^−1^ in phosphate-buffered saline and 2% bovine serum albumin (pH 7.4) for ~45 min in a shaking water bath (220 r.p.m.) at 37 °C. The suspension was filtered and subject to brief, gentle centrifugation (10 s at 100 *g*) before aspiration of adipocytes. After further centrifugation (10 min at 300 *g*), the remaining pellet was resuspended in erythrocyte lysis buffer for 10 min before a final centrifugation step (5 min at 300 *g*).^3^ SVF pellets were stored in 1 ml phosphate-buffered saline:foetal bovine serum:dimethyl sulphoxide (5:4:1) and frozen at a rate of −1 °C min^−1^ using a freezing container (Thermo Scientific) to −80 °C and stored before subsequent analysis by flow cytometry.

### Real-time PCR

Total RNA was extracted from whole adipose tissue using the RNeasy Mini Kit (Qiagen, Crawley, UK). Samples were quantified (Qubit 2.0 fluorimeter; Life Technologies, Paisley, UK) and 2 μg reverse transcribed to cDNA using a High Capacity Reverse Transcription Kit (Applied Biosystems, Warrington, UK). Real-time PCR was performed using a StepOne (Applied Biosystems) with predesigned primers and probes obtained from Applied Biosystems for the measurement of macrophages (CD68: Hs02836816_g1), T lymphocyte populations and subsets (CD3G: Hs00962186_m1; CD4: Hs01058407_m1; CD8A: Hs00233520_m1; FOXP3: Hs01085834_m1; GATA3: Hs00231122_m1; and TBX21: Hs00203436_m1) and for expression of GLUT4 (Hs00168966_m1), IRS2 (Hs00275843_s1), HSL (Hs00193510_m1), leptin (Hs00174877_m1), adiponectin (Hs00605917_m1), MCP-1 (Hs00234140_m1), RANTES (Hs00982282_m1), IP-10 (Hs01124251_g1), IL-6 (Hs00985639_m1), IL-8 (Hs99999034_m1), IL-10 (Hs00961619_m1), IL-1Ra (Hs00893626_m1), TNF-α (Hs99999043_m1), IL-1β (Hs01555410_m1) and IL-18 (Hs00155517_m1). Peptidylpropyl isomerase A (PPIA) was used as an endogenous control.^23^ Results were analysed using the comparative Ct method and expression normalised to an internal calibrator specific to each gene using the formula 2^−▵▵C_t_^, where ▵▵C_t_ is (C_t_ gene of interest−C_t_ PPIA)−lowest ▵C_t_ for gene of interest, and statistical analysis was performed on LN-transformed values.^24^ Data for adipose tissue expression of GCSF (Hs00738432_g1), MIP-1β (Hs01031494_m1) and IFN-γ (Hs00174143) are not shown because they were only detectable in four to eight individuals.

### Analysis of SVF and PBMCs by flow cytometry

Flow cytometry (using the FACSverse; Beckton Dickenson, Erembodegem, Belgium) was used to identify CD4+/CD8+ T lymphocytes (CD45+CD3+ cells) and macrophages/monocytes (CD45+CD14+/CD45+HLA-DR+CD16+ cells) in SVF and PBMCs together with respective levels of activation. Owing to the limited size of SVF samples remaining for analysis by flow cytometry, cells were labelled using a single cocktail comprising the following antibodies: CD4-FITC, CD163-PE, CD14-PerCy5.5, CD8-PE-Cy7, CD69-APC, CD25-APC-Cy7, CD3-V450 and CD45-V500 (Beckton Dickenson). PBMCs were labelled using two separate cocktails to identify monocytes: CD45-V500, CD16-APC, HLA-DR-FITC, CD11b-PeCy5, CD86-PE-Cy7 and T lymphocytes; CD45-V500, CD3-V450, CD4-FITC, CD8-PE-Cy7, CD69-APC, CD25-APC-Cy7 and CD220-PE (Beckton Dickenson).^10^ For an example of gating strategies, see Supplementary Information online.

### Biochemical analysis

Plasma glucose and serum total cholesterol, high-density lipoprotein-cholesterol, alanine aminotransferase activity and triglycerides were measured using commercially available assay kits and analyser (Daytona Rx; Randox, Crumlin, UK). Enzyme-linked immunosorbent assay was used for the measurement of serum insulin (Mercodia, Uppsala, Sweden) and adiponectin (R&D Systems, Abingdon, UK) and both serum and adipose tissue leptin secretion (R&D systems). Adipose tissue secretion of GCSF, MCP-1, IP-10, IL-8, IL-6, IL-10, IL-1Ra, MIP-1β, TNF-α and IL-1β were measured using custom-made kits for use with a fluorescent bead multiplex system (Luminex; Bio-Rad, Hercules, CA, USA) and multiplied by central fat mass (L1–L4) estimated using dual-energy X-ray absorptiometry to predict total central adipose tissue secretion.

### Statistical analysis

All data are presented as mean and standard error of the mean. Comparisons were made between the lean, overweight and obese groups using one-way analysis of variance irrespective of normality.^25^ Relationships between parameters were analysed using Pearson's correlation. Statistical analysis was performed using SPSS version 20 (IBM, Armonk, NY, USA) and *P*<0.05 was considered to be statistically significant.

## Results

### Participant characteristics

Participants in each group differed in terms of physical measures of adiposity and blood concentrations of glucose, insulin and leptin (Table 1).

### Lymphocyte numbers and activation in adipose tissue

**Figure 1:**
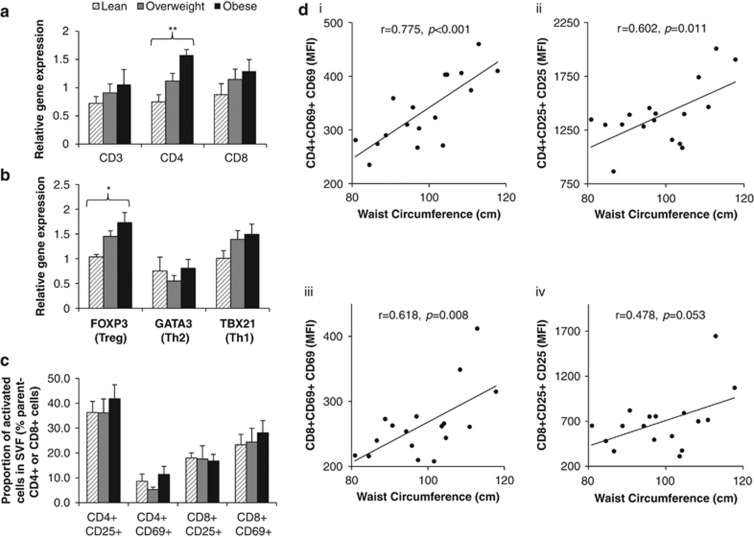
Lymphocyte phenotype and activation in adipose tissue according to levels of central adiposity. (**a**) Relative gene expression of cluster differentiation markers to identify T lymphocytes (*n*=30), (**b**) CD4+ T lymphocyte subsets present within the adipose tissue (*n*=30) and (**c**) proportions of activated T lymphocytes in adipose tissue SVF as a percentage of CD4+ and CD8+ cells measured by flow cytometry (*n*=17). Gene expression data presented as mean 2^−▵▵C_t_^±s.e.m. Effects of adiposity were analysed by one-way analysis of variance (ANOVA), *P<0.05 and **P<0.001. (**d**) (i–iv) Correlations between waist circumference and activation status of CD4+ and CD8+ T lymphocytes in the adipose tissue (*n*=17) were analysed using Pearson's (*R*) correlation; coefficients shown along with significance values. MFI, mean fluorescence intensity.

Gene expression analysis of whole adipose tissue (*n*=30) revealed the presence of T lymphocytes on the basis of CD3, CD4 and CD8 expression (Figure 1a). Relative expression of CD4 was significantly increased with adiposity and further analysis using CD4 lymphocyte lineage markers; FOXP3, (T-regulatory cells), GATA3 (T-helper type 2 cells) and TBX21 (T-helper type 1 cells) revealed an increase only in the relative expression of T-regulatory cell transcripts (Figure 1b).

There was sufficient adipose tissue from 17 of the 30 participants to perform flow cytometry of the SVF to characterise these T lymphocyte populations by both proportion of total cells and activation status. Flow cytometry confirmed the presence of both CD4+ and CD8+ lymphocytes within the CD45+CD3+ population. As a percentage of total cells present in the SVF, CD4+ cells ranged from 0.3 to 4.7% and CD8+ cells ranged from 0.5 to 5.6%, but there were no correlations between cell percentages and measures of adiposity (data not shown).

The lymphocyte subsets were further characterised using activation markers CD69 and CD25. Activated T lymphocytes were assessed according to both the proportion of cells that were activated and their mean level of activation (mean fluorescent intensity of each activation marker). Proportions of activated CD4+ and CD8+ T lymphocytes (CD25+ or CD69+) as a percentage of either total SVF cells or percentage CD4+/CD8+ T lymphocytes (CD45+CD3+ cells) within the SVF were not related to measures of adiposity (Figure 1c). When examining the level of T lymphocyte activation, however, significant correlations were found between central adiposity and the level of expression of CD69 and CD25 on activated CD4+ and CD8+ T lymphocytes (Figure 1d i–iv).

### Macrophage numbers and activation in adipose tissue

**Figure 2:**
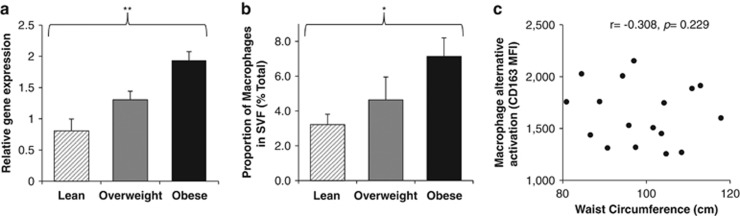
Macrophage numbers and activation in adipose tissue with varying levels of adiposity. (**a**) Relative gene expression of CD68 used to identify macrophages (*n*=30). (**b**) Proportions of macrophages in adipose tissue SVF as a percentage of the total cells (*n*=17). Data are presented as mean 2^−▵▵C_t_^±s.e.m. Effects of adiposity were analysed by one-way analysis of variance (ANOVA), *P<0.05 and **P<0.001. (**c**) Correlations between waist circumference and proportion of macrophages present in the adipose tissue SVF (*n*=17) were analysed using Pearson's (*R*) correlation. MFI, mean fluorescence intensity.

Macrophages were identified using gene expression analysis of CD68 (*n*=30), which was significantly increased with greater levels of adiposity (Figure 2a). Using flow cytometry of the available samples (*n*=17), macrophages (CD45+CD14+) represented 2.9–15.5% of total cells present in the SVF. The proportion of macrophages was positively related to central adiposity (Figure 2b). Flow cytometric analysis of CD163 expression on CD45+/CD14+ cells was used as a measure of macrophage ‘alternative/anti-inflammatory' activation, but this marker was not affected by adiposity (Figure 2c).

### Metabolic and inflammatory properties of adipose tissue

**Figure 3:**
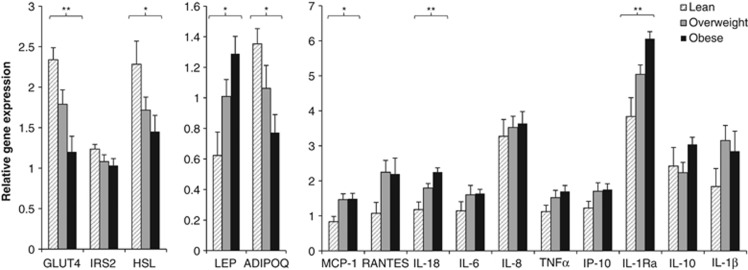
Relative gene expression of parameters related to metabolism, appetite/adiposity and inflammatory cytokines in whole adipose tissue samples with varying levels of adiposity. Data are presented as mean 2^−▵▵C_t_^±s.e.m. with participants classified equally based on waist circumference (*n*=30). Effects of adiposity were analysed by one-way analysis of variance (ANOVA), *P<0.05 and **P<0.005. Note that IL-10 was expressed in three lean, eight overweight and seven obese individuals. IL-6, IL-8 and IL-1β were detected in all overweight and obese individuals but only in eight, six and nine lean individuals, respectively.

To identify potential factors within the adipose tissue that may contribute to this increased T lymphocyte activation and macrophage accumulation with increased adiposity, gene expression and protein secretion from whole adipose tissue was examined. With greater levels of adiposity, relative gene expression of the adiposity-related hormone leptin was increased as expected, with reduced expression of adiponectin, GLUT4 and HSL (Figure 3). The majority of adipose tissue inflammatory cytokines showed a trend towards increased expression with adiposity; however, this only reached statistical significance for the typically pro-inflammatory cytokine IL-18, anti-inflammatory IL-1Ra and MCP-1, a monocyte/macrophage chemoattractant (Figure 3).

**Figure 4:**
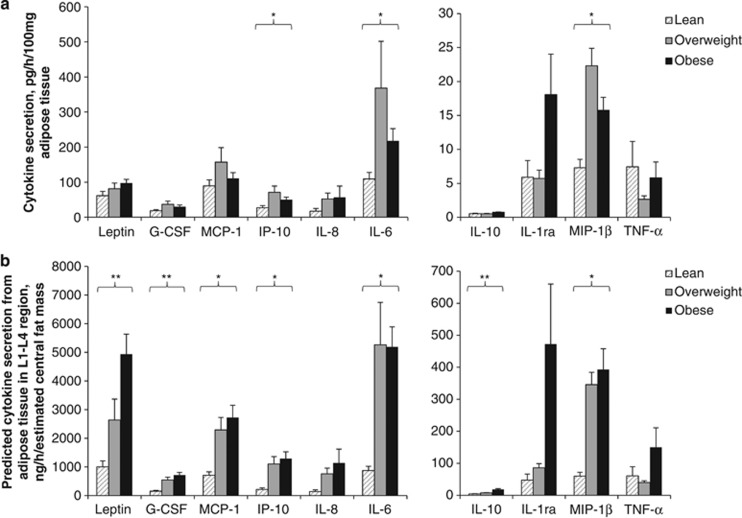
Cytokine secretion by whole adipose tissue explants cultured for 3h with varying levels of adiposity. (**a**) Adipokine secretion normalised per 100 mg ml^−1^ adipose tissue cultured. (**b**) Adipokine secretion multiplied by L1–L4 fat mass to predict total central adipose tissue adipokine secretion. Mean and s.e.m. values were shown for groups based on waist circumference. Effects of adiposity were analysed by one-way analysis of variance (ANOVA), *P<0.05, **P<0.005 and ^$^*P*=0.06 (lean, *n*=8; overweight, *n*=6; obese, *n*=10).

Adipokine secretion from whole adipose tissue explants was determined per 100 mg cultured tissue (Figure 4a) and multiplied by L1–L4 fat mass to predict total central adipose tissue adipokine secretion (Figure 4b). Adjusted adipokine secretion accounts for the profound differences between individuals in absolute levels of adiposity and is thus more representative of secretion *in vivo*. Adjusted adipokine secretion increased with adiposity for the majority of measured adipokines (Figure 4b) and adjusted values better correlated with systemic concentrations (e.g., adipose tissue leptin secretion normalised to L1–L4 fat mass was more strongly correlated with serum leptin than unadjusted leptin secretion: *r*=0.9 vs *r*=0.6, respectively—see Supplementary Information online.

### Relationships between adipose immune cells and inflammatory cytokines/clinical outcomes

A comprehensive unbiased approach was used to investigate relationships between immune cell properties and pro- and anti-inflammatory adipokines in adipose tissue. We also explored relationships between measures of metabolic health that are commonly used in clinical practice. A number of consistent significant positive correlations were observed between levels of T lymphocyte activation and relative levels of gene expression and adipose secretion of IL-18, IL-10, IL-1Ra, leptin and MCP-1 and serum leptin (Table 2). Relationships between the percentage of macrophages in the SVF and adipokines were less consistent at the gene expression and secretion levels, and, instead, appeared to be more closely related to leptin in serum and secretion from adipose tissue, as well as a number of clinical blood markers of metabolic health (Table 2).

### Blood immune cell subsets

To investigate the specificity of these relationships to adipose tissue-resident immune cells, paired blood samples were obtained and PBMCs isolated for characterisation according to cell subset and activation by flow cytometry. None of the correlations observed in the SVF regarding lymphocyte activation with waist circumference were found for paired PBMC samples, indicating that increased activation with increased adiposity is specific to adipose tissue (see Supplementary Information online). Furthermore, in contrast to findings for immune cells in SVF, there were no consistent relationships between PBMC activation and any blood markers of metabolic health.

## Discussion

This is the first study to demonstrate that in men with moderately increased adiposity typical of common overweight and obesity, there is increased activation of adipose tissue-resident T lymphocyte populations. At the whole tissue level, an increase in FOXP3 gene expression with adiposity and reduced pro-inflammatory cytokine production per gram of tissue in obese compared with overweight participants indicates the emergence of potential compensatory mechanisms, possibly through an increase in T-regulatory cells.

### T lymphocytes and their activation in human adipose tissue

Our results from both gene expression and flow cytometry document the presence of CD4+ and CD8+ T lymphocyte populations together with the well-described accumulation of macrophages in human subcutaneous adipose tissue SVF.^1, 2, 3, 4, 5, 10, 11, 12, 13, 14, 15^ Earlier rodent studies investigating the time course of immune cell infiltration with diet-induced obesity suggested that an early increase in CD8+ T lymphocytes may be important and linked to the development of systemic insulin resistance.^12^ Our cross-sectional analysis of immune cells in human adipose tissue with varying levels of adiposity, however, showed no differences in levels of CD8+ T lymphocytes by either gene expression or flow cytometry analysis. Instead, our results showed an accumulation of CD4+ T lymphocytes at the gene expression level. From the 30 participants, there were sufficient cells to perform further analysis by flow cytometry on 17 samples. A substantial fraction of both CD4+ and CD8+ T lymphocytes were in an activated state (CD25+ and/or CD69+) in the men recruited in the present study, as previously reported for women.^10, 16^ Interestingly, although no differences were detected in the *proportion* of activated CD4 and CD8 T lymphocytes within adipose tissue SVF, the level of CD25 and CD69 expression on activated T lymphocytes showed a positive correlation with waist circumference. Importantly, the increased T lymphocyte activation with overweight/obesity was not observed with lymphocytes isolated from paired blood samples—therefore, indicating that this finding is specific to adipose tissue and not a systemic or whole-body response.

In parallel, our gene expression data showed a gradual increase in FOXP3 transcripts with increasing adiposity. The limited number of cells in our SVF samples prevented intracellular staining for FOXP3 or cell sorting of CD25(hi) cells for subsequent *ex vivo* suppression assay. Therefore, although we cannot formally link the increase in FOXP3 transcripts to an increase in T-regulatory cells,^26^ this observation gives a strong indication of a possible increase in T-regulatory cells with increased levels of adiposity. The presence of T-regulatory cells in adipose tissue has been shown in other human studies,^10, 13, 14^ and one other study reported increased T-regulatory cells in men and women with morbid class III obesity.^11^ T-regulatory cells control both adaptive and innate immune responses via their suppressor activity and are therefore vital for immune function and homeostasis.^27^ Human obesity tends to occur over much longer periods of time compared with mouse models where diet-induced obesity develops over just a few weeks.^12^ The more gradual adipose tissue expansion in humans may allow for compensatory mechanisms such as the accumulation of T-regulatory cells as an attempt to limit local inflammation. Indeed, in mouse models, a loss of T-regulatory cells in adipose tissue accompanies the development of insulin resistance,^13, 15^ with gain-of-function experiments improving insulin sensitivity, confirming the potential importance of T-regulatory cells in obesity-related insulin resistance.^13^

### Adipose tissue genotype/phenotype and resident T lymphocyte activation

The presence of relationships between leptin, IL-18, IL-10, IL-1Ra and MCP-1 with levels of CD8+ and CD4+ T lymphocyte activation and proportion of macrophages suggests that there may be complex interactions between the adaptive and innate immune system within adipose tissue. Our results suggest that leptin may be important in adipose tissue T lymphocyte activation, and, indeed, *in vitro* work has shown that leptin enhances T lymphocyte homeostasis/function^28^ with dose-dependent increases in CD4+ and CD8+ expression of CD25 and CD69.^29, 30^ Relationships between CD4+ activation and IL-18 and IL-10 adipose gene expression/secretion in the present study are also of particular interest. IL-18 is a pro-inflammatory cytokine produced by the NLRP3 inflammasome in response to ‘danger signals' and is implicated in the differentiation of CD4+ T lymphocytes into T-helper type 1 cells.^6, 31^ IL-10 conversely is typically anti-inflammatory and indicative of T-helper type 2 cells/T-regulatory lymphocyte differentiation.^6^ This supports the hypothesis that, in addition to pro-inflammatory changes in adipose tissue, there are potential protective compensatory responses involving T lymphocytes.^11, 14^ The absence of relationships between IL-6 or TNF-α with T lymphocyte activation and macrophages may provide further evidence to support this contention. In mouse models of obesity, macrophages switch from an anti-inflammatory M2 phenotype (producing IL-10) to a pro-inflammatory M1 phenotype, which overexpress inflammatory cytokines including IL-6 and TNF-α^32^ and are likely to be a major contributing source of these cytokines in adipose tissue. Human data regarding macrophages suggests that with increased levels of adiposity, macrophage numbers are increased and exhibit an anti-inflammatory phenotype associated with tissue remodelling.^4, 5, 33^ In this context, the absence of relationships between pro-inflammatory cytokines and T lymphocyte activation and macrophage accumulation may not be so surprising, particularly given our focus on modest overweight and obesity. Taken together, these data suggest that the development of a pro-inflammatory phenotype as seen in obesity is associated with adaptive responses and an attempt to develop a protective, more anti-inflammatory profile, which is presumably lost or overcome with either further increases in obesity or a further deterioration of metabolic health.

### Adipose tissue compensation with increased adiposity

Adipose tissue is dynamic and undergoes adaptations in times of both calorie restriction and chronic overnutrition. During chronic energy surplus, adipose tissue expands and regulates expression of proteins related to fatty acid trafficking at the cellular level to prevent increases in fasting blood free fatty acids.^34^ Our observed downregulation of adipose tissue HSL gene expression with increasing adiposity supports this suggestion. This ability of adipose tissue to adapt with its expansion may also extend to regulating secretion of a number of adipokines measured in this study. At the per unit adipose tissue level, secretion of some adipokines including IL-6, IP-10 and MIP-1β was actually reduced in obese individuals (relative to their overweight counterparts). Thus, in the context of modest overweight and obesity, adipose tissue appears to adapt per unit of tissue in an attempt to regulate overall adipokine output to the circulation, although whether this is in any way linked to the downregulation of free fatty acid delivery is far from certain.

### T lymphocytes and systemic markers of metabolic health

In contrast to our findings for adipose tissue-resident macrophages, there were no relationships between T lymphocyte subsets and their activation with systemic measures of health that are routinely used in clinical practice (e.g., homeostasis model assessment-established insulin resistance (HOMA-IR)). This supports findings from another study where no correlations were found between T lymphocyte subsets in subcutaneous adipose tissue and HOMA-IR.^11^ In the present study, the percentage of macrophages in adipose tissue was significantly related to HOMA-IR, high-density lipoprotein (HDL)-cholesterol and ALT. This could indicate that macrophages have a more direct role than lymphocytes in obesity-mediated changes in systemic inflammation/insulin resistance. However, there may be temporal considerations to these comparisons, which make such conclusions difficult. Macrophages may reside in tissues much longer than T lymphocytes and therefore have time to influence/become influenced by changes in local and systemic metabolism and inflammation.^35^ In contrast, effector T lymphocytes can have relatively shorter lifespans and some lymphocyte subsets (e.g., memory T lymphocytes) transit through the tissue before their recirculation.^36^ The dynamic nature of lymphocytes in adipose tissue may make it difficult to draw conclusions about their role based on a snapshot but may also indicate that these fast-changing cell populations represent an exciting opportunity for intervention.

These relationships have been demonstrated across lean to modestly obese middle-aged men, but whether these relationships hold true with further increases in adiposity in women or men of a different age and in people with metabolic complications such as insulin resistance warrants further investigation.

## Conclusion

Following the discovery of immune cells in adipose tissue macrophages have taken centre stage, whereas other cells such as lymphocytes have been somewhat overlooked. The present study demonstrates for the first time that modest adipose tissue expansion is characterised, not by an increase in the proportion of activated T lymphocytes but rather by a stronger state of activation in the T lymphocytes already expressing CD69 and/or CD25. Importantly, this increased activation was not observed in circulating blood T lymphocytes. In addition to positive relationships with pro-inflammatory cytokine production, we show that T lymphocyte activation is also positively related to anti-inflammatory cytokine production at both the gene expression and secretion level—providing further evidence of attempts by adipose tissue and resident T lymphocytes to limit pro-inflammatory output from adipose tissue at least with modestly increased levels of overweight/obesity. From our results, one of the possible mechanisms that could drive this anti-inflammatory compensation is an increased presence of T-regulatory cells. T lymphocytes are therefore likely to have a key role in the regulation of adipose tissue inflammation, and the important adaptations seen even with modestly increased levels of adiposity.

## Figures and Tables

**Table 1:** Participant descriptive statistics

*Classification based on waist circumference*	*Lean,* n*=10*	*Overweight,* n*=10*	*Obese,* n*=10*	*ANOVA* P*-value*
*Physical characteristics*				
Age (years)	43.5±1.7	48.0±1.8	45.2±1.9	0.218
Waist circumference (cm)	87.0±1.4	97.7±0.8	109.4±1.8	<0.001
Body mass index (kg m^−^^2^)	23.6±0.6	26.7±0.4	30.7±0.9	<0.001
Fat mass index (kg m^−^^2^)	4.5±0.3	6.9±0.2	9.5±0.6	<0.001
L1–L4 (%)	19±1.4	30±1.0	37±1.9	<0.001
				
*Fasting metabolic characteristics*				
Leptin (ng ml^−1^)	10.0±1.6	26.6±3.4	40.1±4.4	<0.001
Adiponectin (μg ml^−1^)	9.6±1.3	8.7±1.2	8.5±1.3	0.834
Glucose (mmol l^−1^)	4.4±0.3	4.8±0.3	5.3±0.2	0.043
Insulin (pmol l^−1^)	27.3±4.8	39.6±7.0	59.5±10.1	0.020
HOMA-IR	0.9±0.2	1.4±0.3	2.4±0.4	0.012
Total-cholesterol (mmol l^−1^)	4.6±0.3	5.1±0.3	4.4±0.3	0.267
Triglycerides (mmol l^−1^)	0.9±0.1	1.3±0.2	1.0±0.1	0.123
HDL-cholesterol (mmol l^−1^)	1.3±0.1	1.1±0.1	1.1±0.1	0.091
NEFA (mmol l^−1^)	0.33±0.04	0.48±0.13	0.43±0.05	0.469
ALT (U l^−1^)	18.5±1.3	27.4±2.6	28.5±5.0	0.087

Abbreviations: ALT, alanine aminotransferase; ANOVA, analysis of variance; DEXA, dual-energy X-ray absorptiometry; HDL, high-density lipoprotein; HOMA-IR, homeostasis model assessment-established insulin resistance; L1–L4, central fat mass within the lumbar region L1–L4 as determined by DEXA scan; NEFA, non-esterified fatty acids.

Data presented as mean±s.e.m. Effects of adiposity were analysed by one-way ANOVA (*P*-values shown).

**Table 2:** Associations between adipose tissue immune cell characteristics, blood markers of metabolic health and adipose tissue gene expression and secretion of pro- and anti- inflammatory adipokines

	*Serum*				*mRNA*							*Whole AT protein secretion (3h) adjusted to central fat mass (L1-L4 region)*					
	*Leptin (pg ml^−1^)*	*HOMA-IR*	*Total:HDL chol. ratio*	*ALT (U l^−1^)*	*Leptin*	*IL-6*	*TNF-α*	*MCP-1*	*IL-10*	*IL-1Ra*	*IL-18*	*Leptin*	*IL-6*	*TNF-α*	*MCP-1*	*IL-10*	*IL-1Ra*
*T lymphocytes*																	
CD4+CD69+ MFI	0.557*	0.303	0.219	0.273	0.483*	0.195	0.127	0.596*	0.644*	0.674**	0.742**	0.574*	0.410	0.403	0.633**	0.727**	0.523*
CD4+CD25+ MFI	0.522*	0.409	0.375	0.567*	0.281	0.086	0.363	0.729**	0.576*	0.697**	0.744**	0.645**	0.370	0.156	0.674**	0.596*	0.288
CD8+CD69+ MFI	0.472^$^	0.291	0.264	0.349	0.313	0.020	0.067	0.647**	0.624*	0.795**	0.703**	0.703**	0.351	0.036	0.684**	0.718**	0.222
CD8+CD25+ MFI	0.305	0.200	0.238	0.476^$^	0.161	0.121	0.293	0.757**	0.587*	0.836**	0.609**	0.447^$^	0.233	0.221	0.536*	0.450^$^	0.360
																	
*Macrophages*																	
CD45+CD14+ %total	0.626**	0.485*	0.539*	0.621**	0.449^$^	0.024	0.283	0.383	0.278	0.519*	0.667**	0.769**	0.346	0.097	0.479*	0.671**	0.205

Abbreviations: ALT, alanine aminotransferase; AT, adipose tissue; HDL, high-density lipoprotein; HOMA-IR, homeostasis model assessment-established insulin resistance; IL, interleukin; IL-1Ra, IL-1 receptor antagonist; MCP-1, monocyte chemoattractant protein-1; MFI, mean fluorescent intensity; TNF-α, tumour necrosis factor-α.

Data are presented as Pearson's *R* value (*n*=17; except IL-10 where *n*=13). *P<0.05, **P<0.005, ^$^*P*=0.05-0.1.

## References

[bib1] Weisberg SP McCann D Desai M Rosenbaum M Leibel RL Ferrante AW Jr Obesity is associated with macrophage accumulation in adipose tissue J Clin Invest 2003 112 1796 1808 14679176 10.1172/JCI19246 PMC296995

[bib2] Xu H Barnes GT Yang Q Tan G Yang D Chou CJ Chronic inflammation in fat plays a crucial role in the development of obesity-related insulin resistance J Clin Invest 2003 112 1821 1830 14679177 10.1172/JCI19451 PMC296998

[bib3] Curat CA Miranville A Sengenes C Diehl M Tonus C Busse R From blood monocytes to adipose tissue-resident macrophages: induction of diapedesis by human mature adipocytes Diabetes 2004 53 1285 1292 15111498 10.2337/diabetes.53.5.1285

[bib4] Zeyda M Farmer D Todoric J Aszmann O Speiser M Gyori G Human adipose tissue macrophages are of an anti-inflammatory phenotype but capable of excessive pro-inflammatory mediator production Int J Obes (Lond) 2007 31 1420 1428 17593905 10.1038/sj.ijo.0803632

[bib5] Bourlier V Zakaroff-Girard A Miranville A De Barros S Maumus M Sengenes C Remodeling phenotype of human subcutaneous adipose tissue macrophages Circulation 2008 117 806 815 18227385 10.1161/CIRCULATIONAHA.107.724096

[bib6] Witztum JL Lichtman AH The influence of innate and adaptive immune responses on atherosclerosis Annu Rev Pathol 2014 9 73 102 23937439 10.1146/annurev-pathol-020712-163936 PMC3988528

[bib7] van Oosterhout AJ Bloksma N Regulatory T-lymphocytes in asthma Eur Resp J 2005 26 918 932 10.1183/09031936.05.00011205 16264056

[bib8] Cope AP Schulze-Koops H Aringer M The central role of T cells in rheumatoid arthritis Clin Exp Rheumatol 2007 25 S4 11 17977483

[bib9] Wu H Ghosh S Perrard XD Feng L Garcia GE Perrard JL T-cell accumulation and regulated on activation, normal T cell expressed and secreted upregulation in adipose tissue in obesity Circulation 2007 115 1029 1038 17296858 10.1161/CIRCULATIONAHA.106.638379

[bib10] Duffaut C Zakaroff-Girard A Bourlier V Decaunes P Maumus M Chiotasso P Interplay between human adipocytes and T lymphocytes in obesity: CCL20 as an adipochemokine and T lymphocytes as lipogenic modulators Arterioscler Thromb Vasc Biol 2009 29 1608 1614 19644053 10.1161/ATVBAHA.109.192583

[bib11] Zeyda M Huber J Prager G Stulnig TM Inflammation correlates with markers of T-cell subsets including regulatory T cells in adipose tissue from obese patients Obesity (Silver Spring, MD) 2011 19 743 748 10.1038/oby.2010.123 20508627

[bib12] Kintscher U Hartge M Hess K Foryst-Ludwig A Clemenz M Wabitsch M T-lymphocyte infiltration in visceral adipose tissue: a primary event in adipose tissue inflammation and the development of obesity-mediated insulin resistance Arterioscler Thromb Vasc Biol 2008 28 1304 1310 18420999 10.1161/ATVBAHA.108.165100

[bib13] Feuerer M Herrero L Cipolletta D Naaz A Wong J Nayer A Lean, but not obese, fat is enriched for a unique population of regulatory T cells that affect metabolic parameters Nat Med 2009 15 930 939 19633656 10.1038/nm.2002 PMC3115752

[bib14] Goossens GH Blaak EE Theunissen R Duijvestijn AM Clement K Tervaert JW Expression of NLRP3 inflammasome and T cell population markers in adipose tissue are associated with insulin resistance and impaired glucose metabolism in humans Mol Immunol 2012 50 142 149 22325453 10.1016/j.molimm.2012.01.005

[bib15] Nishimura S Manabe I Nagasaki M Eto K Yamashita H Ohsugi M CD8+ effector T cells contribute to macrophage recruitment and adipose tissue inflammation in obesity Nat Med 2009 15 914 920 19633658 10.1038/nm.1964

[bib16] Bertola A Ciucci T Rousseau D Bourlier V Duffaut C Bonnafous S Identification of adipose tissue dendritic cells correlated with obesity-associated insulin-resistance and inducing Th17 responses in mice and patients Diabetes 2012 61 2238 2247 22596049 10.2337/db11-1274 PMC3425417

[bib17] Health and Social Care Information Centre Health Survey for England 2012 Trend Tables: Adult Trend TablesHealth and Social Care Information, , www.hscic.gov.uk . 18 December 2013.

[bib18] World Health Organisation Waist Circumference and Waist-Hip Ratio: Report of a WHO Expert Consultation Geneva, SwitzerlandWHO 2008

[bib19] Paradisi G Smith L Burtner C Leaming R Garvey WT Hook G Dual energy X-ray absorptiometry assessment of fat mass distribution and its association with the insulin resistance syndrome Diabetes Care 1999 22 1310 1317 10480776 10.2337/diacare.22.8.1310

[bib20] Kelly TL Wilson KE Heymsfield SB Dual energy X-ray absorptiometry body composition reference values from NHANES PLoS One 2009 4 e7038 19753111 10.1371/journal.pone.0007038 PMC2737140

[bib21] Walhin JP Richardson JD Betts JA Thompson D Exercise counteracts the effects of short-term overfeeding and reduced physical activity independent of energy imbalance in healthy young men J Physiol 2013 591 6231 6243 24167223 10.1113/jphysiol.2013.262709 PMC3892474

[bib22] Fain JN Madan AK Hiler ML Cheema P Bahouth SW Comparison of the release of adipokines by adipose tissue, adipose tissue matrix, and adipocytes from visceral and subcutaneous abdominal adipose tissues of obese humans Endocrinology 2004 145 2273 2282 14726444 10.1210/en.2003-1336

[bib23] Neville MJ Collins JM Gloyn AL McCarthy MI Karpe F Comprehensive human adipose tissue mRNA and microRNA endogenous control selection for quantitative real-time-PCR normalization Obesity (Silver Spring, MD) 2011 19 888 892 10.1038/oby.2010.257 PMC4623139 20948521

[bib24] Livak KJ Schmittgen TD Analysis of relative gene expression data using real-time quantitative PCR and the 2(−Delta Delta C(T)) Method Methods 2001 25 402 408 11846609 10.1006/meth.2001.1262

[bib25] Maxwell SE Delaney HD Designing Experiments and Analysing Data: A Model Comparison Perspective Belmont, CA, USAWadsworth 1990

[bib26] Schmetterer KG Neunkirchner A Pickl WF Naturally occurring regulatory T cells: markers, mechanisms, and manipulation FASEB J 2012 26 2253 2276 22362896 10.1096/fj.11-193672

[bib27] Josefowicz SZ Lu LF Rudensky AY Regulatory T cells: mechanisms of differentiation and function Annu Rev Immunol 2012 30 531 564 22224781 10.1146/annurev.immunol.25.022106.141623 PMC6066374

[bib28] Lord GM Matarese G Howard JK Baker RJ Bloom SR Lechler RI Leptin modulates the T-cell immune response and reverses starvation-induced immunosuppression Nature 1998 394 897 901 9732873 10.1038/29795

[bib29] Sanchez-Margalet V Martin-Romero C Santos-Alvarez J Goberna R Najib S Gonzalez-Yanes C Role of leptin as an immunomodulator of blood mononuclear cells: mechanisms of action Clin Exp Immunol 2003 133 11 19 12823272 10.1046/j.1365-2249.2003.02190.x PMC1808745

[bib30] Martin-Romero C Santos-Alvarez J Goberna R Sanchez-Margalet V Human leptin enhances activation and proliferation of human circulating T lymphocytes Cell Immunol 2000 199 15 24 10675271 10.1006/cimm.1999.1594

[bib31] Vandanmagsar B Youm YH Ravussin A Galgani JE Stadler K Mynatt RL The NLRP3 inflammasome instigates obesity-induced inflammation and insulin resistance Nat Med 2011 17 179 188 21217695 10.1038/nm.2279 PMC3076025

[bib32] Lumeng CN Bodzin JL Saltiel AR Obesity induces a phenotypic switch in adipose tissue macrophage polarization J Clin Invest 2007 117 175 184 17200717 10.1172/JCI29881 PMC1716210

[bib33] Fjeldborg K Pedersen SB Moller HJ Christiansen T Bennetzen M Richelsen B Human adipose tissue macrophages are enhanced but changed to an anti-inflammatory profile in obesity J Immunol Res 2014 2014 309548 24741586 10.1155/2014/309548 PMC3987875

[bib34] McQuaid SE Hodson L Neville MJ Dennis AL Cheeseman J Humphreys SM Downregulation of adipose tissue fatty acid trafficking in obesity: a driver for ectopic fat deposition Diabetes 2011 60 47 55 20943748 10.2337/db10-0867 PMC3012196

[bib35] Galli SJ Borregaard N Wynn TA Phenotypic and functional plasticity of cells of innate immunity: macrophages, mast cells and neutrophils Nat Immunol 2011 12 1035 1044 22012443 10.1038/ni.2109 PMC3412172

[bib36] Mora JR von Andrian UH T-cell homing specificity and plasticity: new concepts and future challenges Trends Immunol 2006 27 235 243 16580261 10.1016/j.it.2006.03.007

