# Supplementary material for: The impact of adiposity on adipose tissue-resident lymphocyte activation in humans
Source: Int J Obes (Lond). 2014 Dec 23;39(5):762–9. doi: 10.1038/ijo.2014.195 (PMC4424387; doi:10.1038/ijo.2014.195)
Supplement: Supplementary Figure 1 [file ijo2014195x1.ppt]

## Slide 1
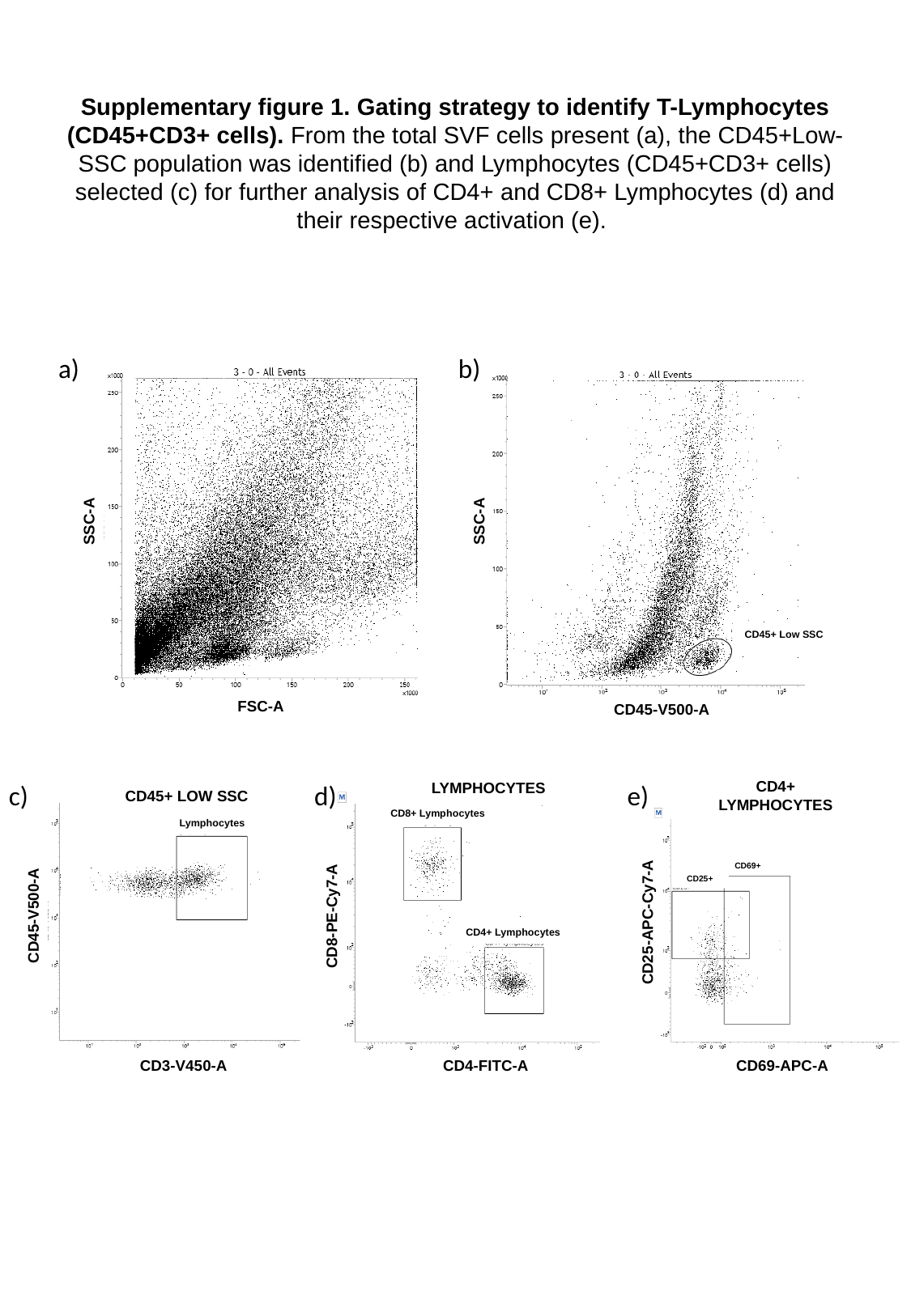

# Supplementary figure 1. Gating strategy to identify T-Lymphocytes (CD45+CD3+ cells). From the total SVF cells present (a), the CD45+Low-SSC population was identified (b) and Lymphocytes (CD45+CD3+ cells) selected (c) for further analysis of CD4+ and CD8+ Lymphocytes (d) and their respective activation (e).
a)
b)
SSC-A
SSC-A
CD45+ Low SSC
FSC-A
CD45-V500-A
CD4+ LYMPHOCYTES
c)
d)
LYMPHOCYTES
e)
CD45+ LOW SSC
CD8+ Lymphocytes
Lymphocytes
CD69+
CD25+
CD45-V500-A
CD8-PE-Cy7-A
CD25-APC-Cy7-A
CD4+ Lymphocytes
CD3-V450-A
CD4-FITC-A
CD69-APC-A
