# Supplementary material for: The impact of adiposity on adipose tissue-resident lymphocyte activation in humans
Source: Int J Obes (Lond). 2014 Dec 23;39(5):762–9. doi: 10.1038/ijo.2014.195 (PMC4424387; doi:10.1038/ijo.2014.195)
Supplement: Supplementary Figure 2 [file ijo2014195x2.ppt]

## Slide 1
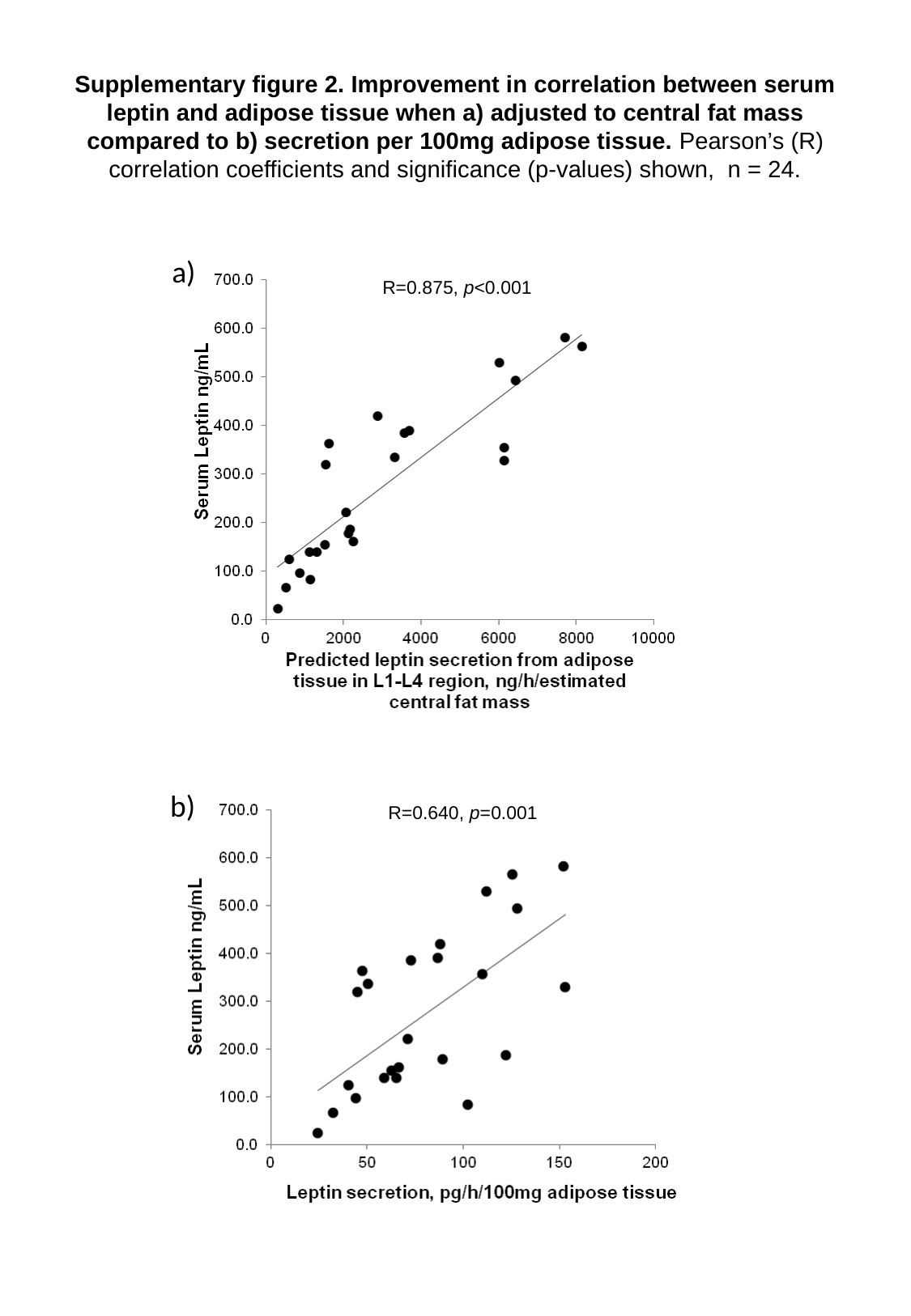

# Supplementary figure 2. Improvement in correlation between serum leptin and adipose tissue when a) adjusted to central fat mass compared to b) secretion per 100mg adipose tissue. Pearson’s (R) correlation coefficients and significance (p-values) shown, n = 24.
a)
R=0.875, p<0.001
b)
R=0.640, p=0.001
