# Supplementary material for: The impact of adiposity on adipose tissue-resident lymphocyte activation in humans
Source: Int J Obes (Lond). 2014 Dec 23;39(5):762–9. doi: 10.1038/ijo.2014.195 (PMC4424387; doi:10.1038/ijo.2014.195)
Supplement: Supplementary Figure 3 [file ijo2014195x3.ppt]

## Slide 1
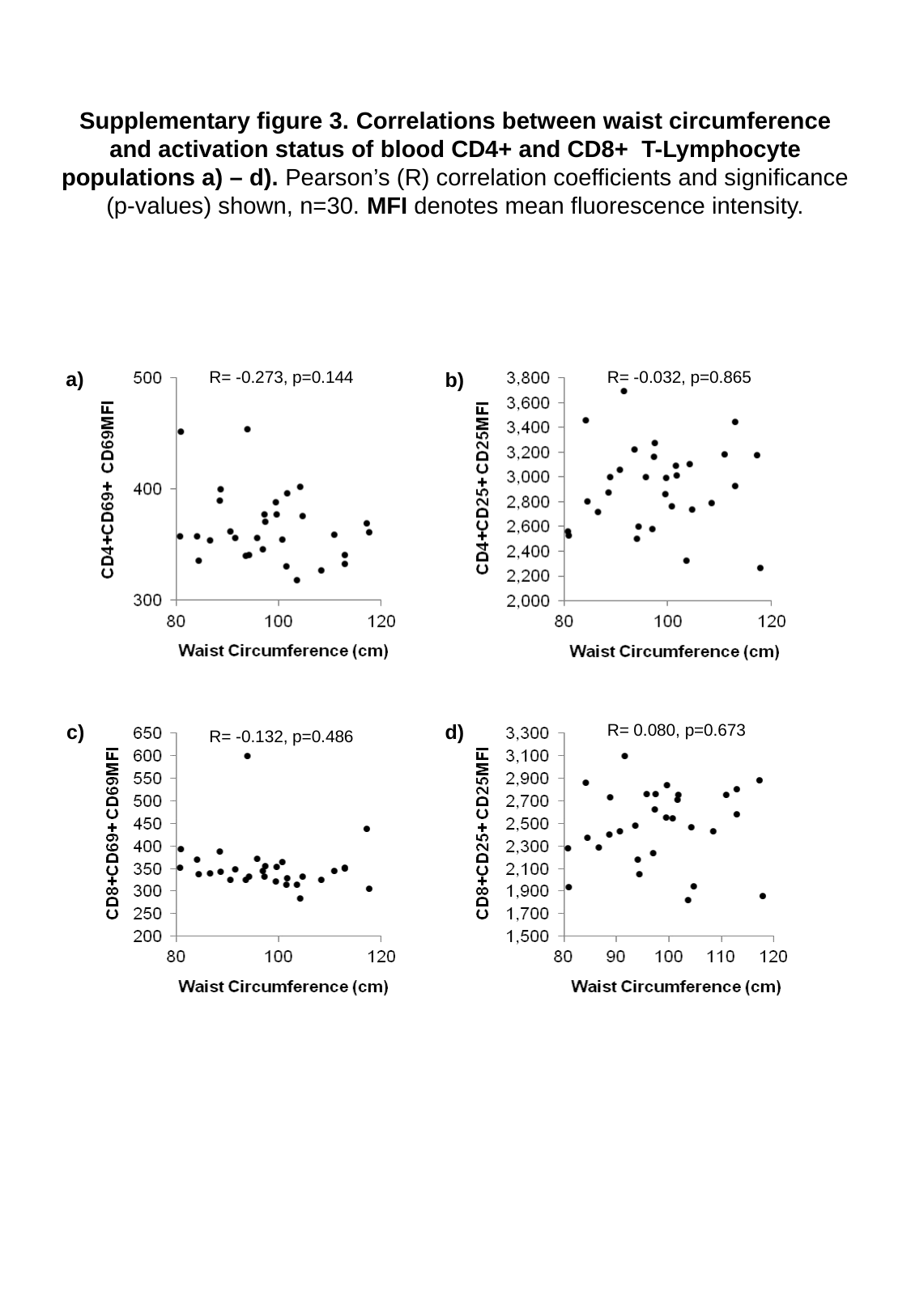

# Supplementary figure 3. Correlations between waist circumference and activation status of blood CD4+ and CD8+ T-Lymphocyte populations a) – d). Pearson’s (R) correlation coefficients and significance (p-values) shown, n=30. MFI denotes mean fluorescence intensity.
a)
R= -0.273, p=0.144
R= -0.032, p=0.865
b)
c)
d)
R= 0.080, p=0.673
R= -0.132, p=0.486
